# The Effects of High Altitudes upon the Efficiency of Aviators

**Published:** 1918

**Authors:** 


### AVIATION

**The Effects of High Altitudes upon the Efficiency of Aviators.** By Major E. G. SEIBERT, M. R. C. Abs. from *The Military Surgeon*, February 1918.

It is a well established fact that pilots show marked evidences of nerve breakdown or instability in greater or less time according to the individual. As the war has progressed, the altitudes to which pilots are forced to go have become more and more pronounced. The percentage of oxygen in the atmosphere remains constant, irrespective of the height. The partial pressure, or tension, however, varies and diminishes in a somewhat progressively proportional manner with the decrease in barometric pressure. It is the oxygen tension in the air cells of the lungs which is most important in maintaining the respiratory process. As the transfer of oxygen from the alveolar air to the blood depends, in part, upon the oxygen tension, it can readily be seen why oxygen want should promptly ensue on reaching high altitudes.

One other factor in this transfer of the oxygen to the blood is the activity of the alveolar epithelium, or cells lining the alveoli. This explains why some may go to these heights with less symptoms resulting than others.

In altitudes above 16,000 feet, where the barometric pressure is half the sea-level pressure and the oxygen tension reduced to one-fourth or less, the blood is quickly deprived of the necessary oxygen to carry on the body process, and allows of the accumulation of those substances which, sooner or later, bring on pronounced intoxication. This intoxication is particularly marked in the nervous system. Its effects are peculiar and especially dangerous to the aviator. Extraordinary impairment of judgment is extremely characteristic, though the person himself is quite confident that his mind is absolutely clear. Perception is more affected than sensation. Other effects are panting, difficulty in concentrating attention, headache and fatigue after landing, vertigo, vomiting, noises and pains in the ears.

Prime physical fitness is not always a guarantee that the subject will be free from nerve instability. This fact points very clearly to the necessity for selection of the men who have been in training for overseas work.

